# Effects of poly(vinyl alcohol) blending with Ag/alginate solutions to form nanocomposite fibres for potential use as antibacterial wound dressings

**DOI:** 10.1098/rsos.211517

**Published:** 2022-03-30

**Authors:** Srdjan Vidovic, Jasmina Stojkovska, Milan Stevanovic, Bojana Balanc, Maja Vukasinovic-Sekulic, Aleksandar Marinkovic, Bojana Obradovic

**Affiliations:** ^1^ Faculty of Technology and Metallurgy, University of Belgrade, 11000 Belgrade, Serbia; ^2^ Innovation Center of the Faculty of Technology and Metallurgy, 11000 Belgrade, Serbia

**Keywords:** Fourier transform infrared spectroscopy, antibacterial activity, silver release, swelling, mechanical properties

## Abstract

In this work, nanocomposite fibres and microfibres based on alginate and poly(vinyl alcohol) (PVA) with silver nanoparticles (AgNPs) were produced and characterized for potential application as antibacterial wound dressings. PVA/Ag/Na-alginate colloid solution was used for the preparation of the fibres by a simple extrusion technique followed by freezing–thawing cycles. UV–Visible spectroscopy confirmed successful preservation of AgNPs in fibres while Fourier transform infrared spectroscopy has shown a balanced combined effect on the Ca-alginate spatial arrangement with the addition of both AgNPs and PVA. The presence of PVA in fibres induced an increase in the swelling degree as compared with that of Ag/Ca-alginate fibres (approx. 28 versus approx. 14). Still, the initially produced PVA/Ca-alginate fibres were mechanically weaker than Ca-alginate fibres, but after drying and rehydration exhibited better mechanical properties. Also, the obtained fibres released AgNPs and/or silver ions at the concentration of approximately 2.6 µg cm^−3^ leading to bacteriostatic effects against *Staphylococcus aureus* and *Escherichia coli*. These results are relevant for practical utilization of the fibres, which could be stored and applied in the dry form with preserved mechanical stability, sorption capacity and antibacterial activity.

## 1. Introduction

Application of wound dressings is essential in treatments of chronic, non-healing wounds, which present a serious public health problem due to the constant rise in the number of affected patients while traditional wound dressings do not promote healing [1]. In developed countries, the prevalence rate for chronic wounds is 1% to 2% of the general population [2] where infection is a common problem preventing healing of these wounds [3].

One of the possible solutions is utilization of advanced wound dressings that should effectively regulate moisture levels in wounds, maintain a stable temperature, protect the wound from infection and remove the dead tissue along with providing easy removal and low frequency of necessary dressing changes. Currently, numerous research studies focus on the development of multifunctional wound dressings including bactericidal products targeting multidrug-resistant bacteria [4]. Specifically, due to the rise in the number of antibiotic-resistant bacteria, the current focus is on finding alternative antimicrobials such as silver and silver-based compounds [5–7] and natural products including honey [8,9], essential oils [10] and chitosan [11]. Silver is one of the most investigated antimicrobial agents today, especially in the form of nanoparticles, due to their powerful antimicrobial activity and broad inhibitory biocide spectra for variety of microbes [6,12].

In recent years, alginate hydrogels have become widely used in advanced wound care products owing to high water sorption capacity and ability to rehydrate the tissue. On the other hand, these hydrogels stimulate collagen type I synthesis, keratinocyte differentiation and fibroblast proliferation, while reducing inflammatory reactions, leading to enhanced tissue regeneration [13–15]. Alginate hydrogels can be easily produced in different shapes (microbeads, microfibres and discs), which were shown to be suitable for immobilization of different active components including silver nanoparticles (AgNPs), honey and activated charcoal microparticles [16–19]. Ca-alginate hydrogels with incorporated AgNPs were shown to exhibit antibacterial activity [20,21], as well as to promote wound healing in animal models [22]. However, these hydrogels display weak mechanical properties, thus demanding more frequent changes [23]. Mechanical properties of alginate hydrogels could be improved by the addition of other polymers such as poly(vinyl alcohol) (PVA) [24,25], gelatin [26] and chitin/chitosan [27]. PVA is a synthetic polymer that can form hydrogels by chemical cross-linking [28,29] as well as physical cross-linking by radiation [30], cast-drying [31] and freezing–thawing cycles [32]. These hydrogels are hydrophilic, biocompatible, exhibiting good mechanical properties and were shown to be suitable for biomedical applications such as in articular cartilage replacement, wound dressings and controlled drug-releasing devices [33–35]. PVA blends with Na-alginate cross-linked by repeated cycles of freezing and thawing were investigated for wound dressing applications with the aim to use beneficial properties of both polymers i.e. favourable mechanical properties of PVA and improved biological properties obtained by Na-alginate addition [36,37] as well as better release profiles of immobilized active agents [38]. On the other hand, blends of PVA and Na-alginate could be gelled by the addition of Ca^2+^ ions, only [39], while strong PVA/Ca-alginate hydrogels were obtained by repeated freezing–thawing cycles followed by alginate gelation by the addition of a concentrated CaCl_2_ solution forming dual-physical double-network [40]. In these hydrogels, sparsely hydrogen-bonded PVA served as a ductile matrix whereas the densely ionically cross-linked alginate served as a rigid skeleton [40].

In the present study, we aimed to apply gelation of both polymers (PVA and alginate) but to produce hydrogels in the form of fibres and microfibres containing AgNPs to be suitable as potential antimicrobial wound dressings. Thus, the specific aims were to (i) develop a simple procedure for preparation of fibres and microfibres, (ii) investigate the influence of PVA on the resulting PVA/alginate hydrogel properties, and (iii) assess antibacterial effects of the obtained fibres against one model Gram-positive (*Staphylococcus aureus)* and one model Gram-negative (*Escherichia coli)* bacteria.

## 2. Material and methods

### 2.1. Materials

Sodium alginate was purchased from Acros Organics (A0328671, Geel, Belgium), ammonium hydroxide (25 wt%) from NRK Inzenjering (Belgrade, Serbia), sodium metasilicate (Maxima, Lucani, Serbia), 1,2,3-benzotriazole from Applichem (A4727, Darmstadt, Germany), monoethylene glycol (MEG) from Centrohem (Belgrade, Serbia) and nitric acid (65%) from Zorka Pharma (Sabac, Serbia). Low-viscosity sodium alginate (A-2158), PVA (hot water soluble, P1763), sodium tetraborate decahydrate (W302600), sodium chloride (S5886), calcium nitrate tetrahydrate (31218) and sodium citrate dehydrate (W302600) were supplied from Sigma-Aldrich Chemie GmbH (Germany).

### 2.2. Synthesis of silver nanoparticles

AgNPs were synthesized in aqueous solutions of Na-alginate by electrochemical reduction as previously described [41]. The electrochemical synthesis was performed galvanostatically in aqueous alginate solution (0.1 M KNO_3_, 3.9 mM AgNO_3_ and 2% w/v Na-alginate), at the current density of 50 mА cm^−2^ and implementation time of 10 min. As it was obtained in the previous studies [16], slight polymer deposition was noted on the counter electrode resulting in the final alginate concentration of 1.81 ± 0.08% w/v.

### 2.3. Production of nanocomposite fibres and microfibres

**Figure 1.**
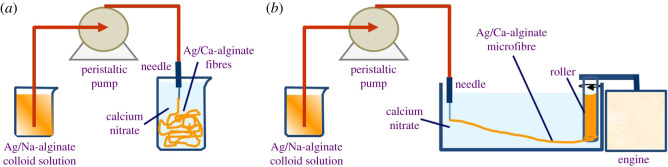
Experimental set-up for Ag/Ca-alginate fibre production by extrusion of Ag/Na-alginate solution through a needle immersed in a gelling bath containing Ca^2+^ (0.18 M) by using a peristaltic pump: (*a*) insoluble fibres were formed by simple extrusion only; (*b*) microfibres were formed by extrusion into the gelling bath followed by stretching and wounding on a rotating roller.

Ag/Ca-alginate fibres were produced by using a simple extrusion technique (figure 1), as described previously [22]. In brief, the Ag/Na-alginate colloid solution was extruded at the flow rate of 14.3 cm^3^ min^−1^ through a blunt edge, stainless steel needle (19G) immersed in a gelling bath by using a peristaltic pump (Behr Labour-Technik, Germany). The gelling solution was 3% w/v calcium nitrate tetrahydrate. Due to the exchange of Na^+^ with Ca^2+^, the liquid stream solidified in the gelling bath, thus forming insoluble fibres. After the completion of gelation (2 h), the obtained fibres were washed in deionized water.

In order to obtain microfibres (less than 1 mm in diameter), a roller (18 mm in diameter) rotating at a constant speed was positioned in the gelling bath (figure 1*b*). Extrusion of the Ag/Na-alginate solution was carried out under the same conditions and the obtained insoluble fibres were wound on the roller and stretched to form microfibres. After 2 h, the microfibres were washed in deionized water.

Pure Ca-alginate fibres and microfibres as controls were produced by the same extrusion techniques.

PVA/Ag/Ca-alginate fibres and microfibres were also produced by the same technique, followed by freezing–thawing cycles. Specifically, PVA powder was dissolved in hot deionized water (80°C) under constant stirring until a clear solution was obtained with the PVA concentration of 17% w/v. The obtained solution was then mixed with Ag/Na-alginate colloid solution in the desired ratio (1 : 2) in order to obtain final solutions with the following composition: 5.7% w/v PVA, 1.27 ± 0.08% w/v Na-alginate and AgNPs at 2.6 mM nominal silver concentration. Then, the solution was extruded as described above. The obtained fibres and microfibres were transferred to a container with a cooling solution composed of 92% w/v MEG, 1.5% w/v sodium metasilicate, 2% w/v sodium tetraborate decahydrate and 0.35% w/v benzotriazole in water [42] and submitted to four freezing–thawing cycles according to the modified procedure for preparation of cryogels [43]. Freezing was performed at −20°C for 20 h, while thawing was carried out at +4°C for 4 h. After the process completion, the fibres were washed in deionized water.

Pure PVA fibres were produced by extrusion of 5.7% w/v PVA solution through a blunt edge needle (19G) immersed in the cold (−20°C) cooling solution. The liquid stream immediately solidified in the gelling bath forming fibres, which were further submitted to four freezing–thawing cycles. The conditions of freezing–thawing cycles were the same for all fibres containing PVA.

Dry fibres and microfibres were obtained by spreading half of all produced fibre and microfibre samples on glass dishes and drying in an oven at 50°C until constant weight.

### 2.4. Characterization of nanocomposite fibres

#### 2.4.1. Swelling properties

Dried PVA/Ag/Ca-alginate fibres (approx. 2 g) were immersed in approximately 40 cm^3^ of physiological saline solution (0.9% w/v NaCl) at room temperature and weighed until reaching equilibrium. Ag/Ca-alginate fibres served as a control. For each time point, the immersed samples were gently dried, weighed and then returned into the solution again. All experimental time points were performed in triplicate.

The swelling degree (*q*) was calculated as

2.1 $$q=\frac{W_{S}-W_{d}}{W_{d}},$$

where *W_S_* and *W_d_* are the weights of the swollen and the initial dried fibres, respectively.

#### 2.4.2. Mechanical properties

Tensile stress at break (*σ*_max_) values of Ca-alginate (approx. 700 µm in diameter) and PVA/Ca-alginate (approx. 1000 µm in diameter) fibres were measured by using a Universal Testing Machine, AG–X Plus (Shimadzu, Japan). The initial grip separation was set at 30 mm and the test speed was set at 10 mm min^−1^. The fibres were sprayed with distilled water initially and every 15 s during the mechanical testing experiment. Young's modulus (E) was calculated by using the initial slope (3% of deformation) of the obtained stress–strain curves. All measurements were done at least in triplicates, at room temperature.

#### 2.4.3. Antibacterial activity of PVA/Ag/Ca-alginate fibres

Antibacterial activity of wet and dried PVA/Ag/Ca-alginate fibres was assessed against *Staphylococcus aureus* TL (culture collection, Faculty of Technology in Leskovac, University of Nis, Leskovac, Serbia) and *Escherichia coli* ATCC 25922, as model bacteria strains for Gram-positive and Gram-negative species, respectively, as previously described [16,20]. PVA/Ag/Ca-alginate fibres obtained from the colloid solution containing 5.67% w/v PVA, 1.27 ± 0.08% w/v Na-alginate and AgNPs at 2.6 mM nominal silver concentration were produced sterile as described above, and a portion of the fibres was dried in an oven at 50°C until constant weight. In each flask, 4 g of wet fibres or approximately 80 mg of corresponding dried fibres equivalent to the same wet weight (i.e. 4 g) were added followed by the addition of 10 cm^3^ of sterile Luria–Bertani (LB) broth (10 g dm^−3^ tryptone, 5 g dm^−3^ yeast extract, 10 g dm^−3^ NaCl) and aliquots of 0.2 cm^3^ precultured bacterial suspensions (not older than 18 h), so that the initial number of bacterial cells in the broth was approximately 10^6^ CFU cm^−3^. The flasks were incubated in a shaking water bath at 37°C, 125 r.p.m. for 24 h and bacterial cultures in LB broth without PVA/Ag/Ca-alginate fibres were used as controls. After 1 h and 24 h of incubation, 0.1 cm^3^ of liquid sample was aseptically withdrawn from each flask and the number of viable cells was determined by the pour plate method on LB agar medium. After 24 h of incubation at 37°C, the formed colonies were counted. The experiments were performed in duplicates and results are expressed as CFU cm^−3^.

In order to determine the released silver concentration during antibacterial activity studies, a parallel experiment was performed in physiological saline solution using wet and dried PVA/Ag/Ca-alginate fibres under the same conditions. The experiment lasted for 24 h and was performed in triplicates.

### 2.5. Analytical methods

#### 2.5.1. UV–Visible spectroscopy

UV–Vis spectroscopy (UV-3100 spectrophotometer, MAPADA, Shanghai, China) was used to confirm the presence of AgNPs in colloid solutions and hydrogels after the dissolution of wet or dried fibres. The fibres were dissolved in 2.28% w/v sodium citrate solution similarly as Ag/alginate microbeads described previously [16]. In brief, wet hydrogel weight of 0.1 g was dissolved in 2.9 cm^3^ of the solution and the dry hydrogel weight of 0.01 g in 6 cm^3^.

#### 2.5.2. Optical microscopy

In order to determine diameters of the obtained fibres, we used optical microscopy (Olympus CX41RF, Tokyo, Japan) with the image analysis program ‘CellА’ (Olympus, Tokyo, Japan). At least 10 fibres were measured to obtain the mean diameter values.

#### 2.5.3. Fourier transform infrared spectroscopy

The IR spectra of dry Ca-alginate, Ag/Ca-alginate, PVA, PVA/Ca-alginate and PVA/Ag/Ca-alginate fibres in the form of KBr pallets were recorded in the transmission mode between 400 and 4000 cm^−1^ using a BOMEM MB100 spectrophotometer (Hartmann & Braun, Canada), while the IR spectrum of Na-alginate powder was recorded in the transmission mode between 550 and 4000 cm^−1^ by using a Thermo Scientific Nicolet iS10 FT-IR spectrometer (Thermo Fisher Scientific, Waltham, MA) with a resolution of 4 cm^−1^, operating in ATR mode.

#### 2.5.4. Silver concentration

The colloid solution containing 5.67% w/v PVA, 1.27 ± 0.08% w/v Na-alginate and AgNPs at 2.6 mM nominal silver concentration was used to produce PVA/Ag/Ca-alginate fibres as described above. To obtain dried fibres, a portion of the fibres was dried in an oven at 50°C until the constant weight. In each flask, 4 g of wet fibres or approximately 80 mg of corresponding dried fibres equivalent to the same wet weight (i.e. 4 g) was added followed by the addition of 10 cm^3^ of physiological saline solutions. The flasks were incubated in a shaking water bath at 37°C and 125 r.p.m. After 24 h, the silver content released in physiological saline solutions was determined according to the procedure previously described [20]. Briefly, NH_4_OH solution (25 wt%) was added in excess (0.5 cm^3^ of the NH_4_OH solution per 1 cm^3^ of the saline solution) directly into the flasks in which the experiments were performed in order to dissolve precipitated AgCl. Concentrations of Ag^+^ in all resulting solutions were then determined at four-digit accuracy by atomic absorption spectroscopy (AAS) by using a Perkin Elmer 3100 spectrometer (Perkin Elmer, MA, USA).

It should be noted that the total silver content released in physiological saline solution determined by the described procedure included released Ag^+^, as well as AgCl [20].

#### 2.5.5. Statistical analysis

Statistical analysis was carried out by the one-way ANOVA using the Microsoft Office Excel software (Microsoft Corp., USA). Values of *p* < 0.05 were considered significant.

## 3. Results and discussion

### 3.1. Production of nanocomposite fibres

**Figure 2.**
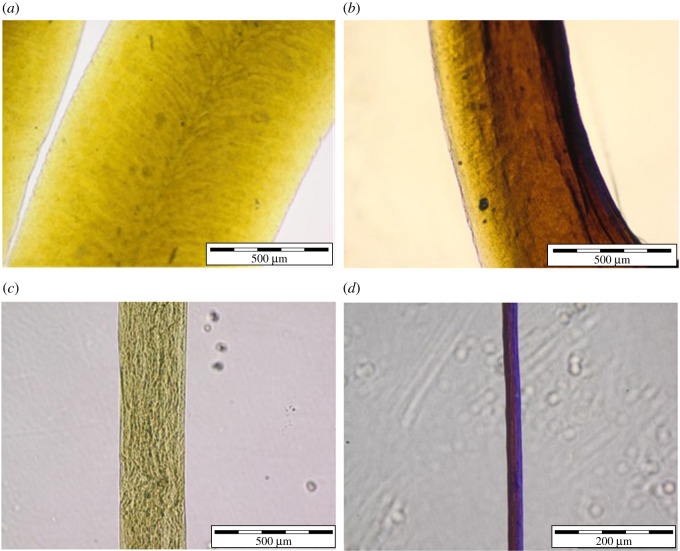
Optical micrographs of (*a*) wet PVA/Ag/Ca-alginate fibre (scale bar: 500 µm); (*b*) dry PVA/Ag/Ca-alginate fibre (scale bar: 500 µm); (*c*) wet PVA/Ag/Ca-alginate microfibre (scale bar: 500 µm); (*d*) dry PVA/Ag/Ca-alginate microfibre (scale bar: 200 µm).

Nanocomposite PVA/Ag/Ca-alginate fibres and microfibres were produced by extrusion of the solution containing 5.7% w/v PVA, 1.27 ± 0.08 w/v Na-alginate and AgNPs at 2.6 mM nominal silver concentration as described in §2.3. The resulting wet fibres and microfibres had mean diameters of 1110 ± 80 µm and 310 ± 20 µm, respectively (figure 2). Both hydrogel forms retained approximately 2% of the initial wet weight upon drying until the constant weight. The obtained dried fibres and microfibres had mean diameters of 470 ± 90 µm and 30 ± 5 µm, respectively (figure 2).

**Figure 3.**
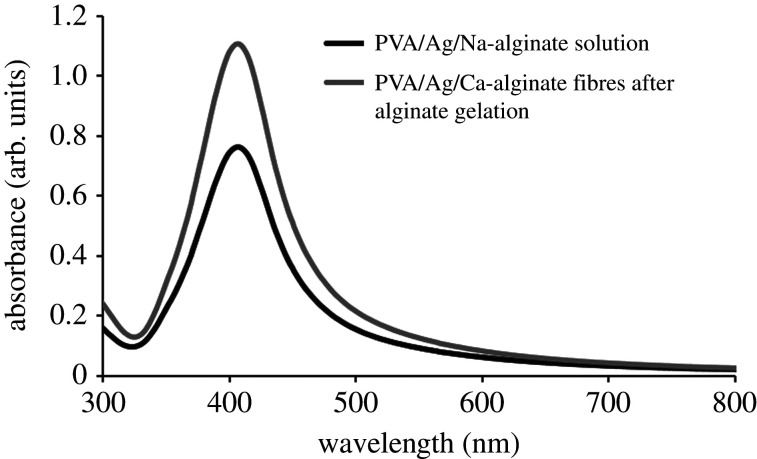
UV–Vis absorption spectra of the initial PVA/Ag/Na-alginate solution (2.6 mM nominal silver concentration) and resulting fibres produced after gelling of alginate (data represent average of *n* ≥ 3; standard deviations (less than or equal to 20%) are omitted from the graph for clarity).

The presence of AgNPs in both PVA/Ag/Ca-alginate hydrogel forms (i.e. fibres and microfibres) was confirmed by UV–Vis spectroscopy. Figure 3 presents representative UV–Vis absorption spectra of the starting PVA/Ag/Na-alginate colloid solution and dissolved corresponding nanocomposite fibres after the gelation of alginate only. Both spectra have shown the absorption maximum at 405 nm confirming that nanoparticles remained stable without forming aggregates during the fibre production process. In addition, the value of the absorbance maximum increased after nanocomposite fibre gelation as compared with the initial PVA/Ag/Na-alginate colloid. A similar result was obtained previously in Ag/alginate microbead production [44], which was explained by the gel contraction during gelation. The increase in silver concentration in PVA/Ag/Na-alginate fibres was confirmed by measurements of silver concentration upon dissolution by AAS amounting to 3.06 ± 0.44 mM in contrast with the total silver concentration of 2.6 mM in the initial colloid solution.

### 3.2. Fourier transform infrared analysis

**Figure 4.**
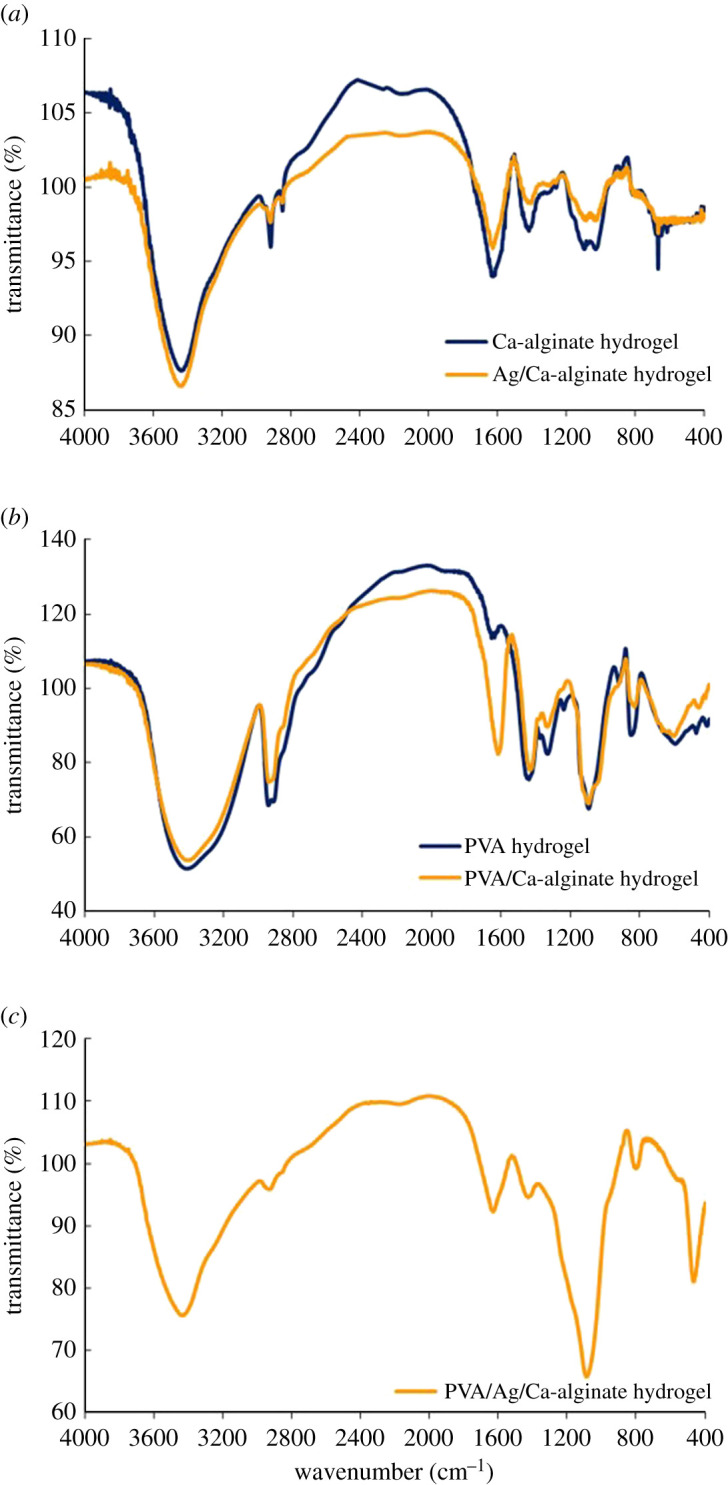
FTIR spectra of (*a*) Ca-alginate and Ag/Ca-alginate fibres; (*b*) pure PVA and PVA/Ca-alginate fibres; (*c*) PVA/Ag/Ca-alginate fibres.

Interactions of the alginate cross-linked structure with AgNPs and PVA macromolecules in the produced nanocomposite fibres were investigated by using Fourier transform infrared (FTIR) spectroscopy. FTIR spectra of all samples are presented in figure 4, while the assignments of the absorption bands are given in table 1. The spectrum of sodium alginate is shown in the electronic supplementary material, figure S1.

**Table 1.** Assignments of the absorption bands for Na-alginate (NaAlg), Ca-alginate (CaAlg), Ag/Ca-alginate (Ag/CaAlg), PVA, PVA/Ca-alginate (PVA/CaAlg) and PVA/Ag/Ca-alginate (PVA/Ag/CaAlg) fibres.

wavenumber, cm^−1^						
NaAlg	CaAlg	Ag/CaAlg	PVA	PVA/CaAlg	PVA/Ag/CaAlg	assignment
3246	3441	3441	3414	3404	3439	*ν*(OH)
	2920	2922	2941	2939	2932	*ν*_as_(CH_2_)
	2851	2851	2912	2914		*ν*_s_(CH_2_)
1594	1628	1630	1655^a^	1618	1630	*ν*_as_(COO^−^)
1405	1420	1408	1439^b^	1431	1427	*ν*_s_(COO^−^)
	1319, 1261	1304, 1261	1331, 1238	1331		*δ*(C-O-H)^c^
1023	1097, 1032	1090, 1030	1094	1095	1090	*ν*(C-OH)
946	887, 815	885	918, 851	829	804	*ν*(C-C)
	669	667	596	604		C-O internal rotation
						C-C-O bending
						C-C-H bending

^a^(OH) contribute to the intensity of observed peak.

^b^*δ*_as_(CH_2_) contribute to the intensity of observed peak.

^c^*δ*_s_(CH_2_) contribute to the intensity of observed peak.

In the spectra of Ca-alginate and Ag/CaAlg fibres (figure 4*a*), PVA and PVA/CaAlg (figure 4*b*) and PVA/Ag/CaAlg (figure 4*c*), the strong and broad band, characteristic for stretching vibrations of –OH participating in hydrogen bonding interactions, is found in the region of 3200–3600 cm^−1^ (table 1). The position of *ν*(OH) vibrations is influenced by the strength of intermolecular hydrogen bonding between secondary hydroxyl groups and properties (surface functionalities, charges, voluminosity/geometry, etc.)/other interactions of the present constituents.

Sharp and low-intensity peaks originating from C-H stretching vibrations were noticed at approximately 2920 and approximately 2851 cm^−1^ in the spectrum of Ca-alginate. Asymmetric and symmetric stretching vibrations of –COO^−^ groups were detected at 1628 and 1420 cm^−1^, respectively. In comparison, the spectrum of sodium alginate (supporting information, electronic supplementary material, figure S1) also displayed absorption peaks at 1594 and 1405 cm^−1^, respectively. Small shifts of both vibrations to higher frequencies indicate that the exchange of sodium ion causes appropriate C-O bond force strengthening in the calcium carboxylate structure. Absorption bands originating from polysaccharide structure appeared at 1319, 1261, 1097 and 1032 cm^−1^ and are attributed mostly to *δ*(C-O-H) and *ν*(C-OH) vibrations, while the peaks at 815 and 887 cm^−1^ are assigned to C-C stretching vibrations.

The FTIR spectrum of the Ag/Ca-alginate fibres is also shown in figure 4*a*. Small observable changes in relation to the Ca-alginate spectrum were noticed in terms of the position and shape of bands assigned to stretching vibrations of carboxylate groups. The peak of asymmetric vibration appeared at a similar position and the symmetric one is shifted towards a lower value, i.e. 1408 cm^−1^, with respect to that of Ca-alginate fibres. This finding suggests that incorporation of AgNPs contributes to low interference with cohesive bonding in the Ca-carboxylate structure.

In the FTIR spectrum of poly(vinyl alcohol) (PVA), fibres (figure 4*b*) peaks of the OH stretching and bending vibrations appeared at 3414 and 1655 cm^−1^, respectively. Asymmetric and symmetric methylene group vibrations were found at 2941 and 2912 cm^−1^, respectively. The IR signals at 1439, 1094 and at around 851 cm^−1^ are attributed to CH_2_ bending, C-O-H stretching and C-C stretching vibrations, respectively. PVA crystallinity is signified by the absorption peak at 1141 cm^−1^, which arises from a C-C stretching mode and increases with an increase in the degree of PVA crystallinity [45].

Significant changes in the spectrum of PVA/Ca-alginate were found in relation to that of PVA fibres. The strong hydroxyl band (figure 4*b*), in the region of 3200–3600 cm^−1^, remains similar to the one of pure PVA fibres. The peaks assigned to asymmetric and symmetric stretching vibrations of carboxylate groups (COO^–^) appeared at 1618 and 1431 cm^−1^, respectively. Intermolecular interactions between functional groups present in structures of both components are of moderate intensities in relation to strong electrostatic attraction in the Ca-alginate structure. Otherwise, opposite effects were found in relation to the spectrum of Ag/Ca-alginate fibres. The peak related to asymmetric carboxylate vibration is slightly shifted to the lower frequency and symmetric stretching is shifted towards a higher value, as compared with Ag/CaAlg (table 1). Such trend suggests that blending of PVA and Ca-alginate contributes to the opposite behaviour in relation to introduction of AgNPs due to contribution of pronounced hydrogen bonding capability of OH groups causing changes in bond strengths in an opposite manner to those induced by AgNPs.

The addition of AgNPs to PVA/Ca-alginate (figure 4*c*) causes an appropriate change in the vibration modes in relation to those of Ag/Ca-alginate (figure 4*a*) and PVA/Ca-alginate (figure 4*b*) fibres. The FTIR spectra showed that the intensity/shape of observed bands is significantly changed, while bands shifting are of low relevance for interpretation of the spectra. The same wavenumber for asymmetric carboxylate stretching vibrations was found for both PVA/Ag/Ca-alginate and Ag/Ca-alginate (table 1). On the other hand, the band corresponding to symmetric vibrations showed a shift to a higher value, i.e. 1427 cm^−1^, in comparison with that of Ag/Ca-alginate, and a slightly lower value with respect to that of PVA/Ca-alginate (table 1). The obtained results indicate that balanced contributions of the effects of AgNPs and PVA addition on the spatial arrangement and overall interactions in the composite structure are operative at different extents. Intensity of the absorption band attributed to C-OH stretching is the highest for this sample and slightly shifted to a lower wavenumber of 1090 cm^−1^. These results indicate that incorporation of AgNPs cause appropriate formation of internally arranged structure with a pendant fragment of PVA moiety at the surface of the PVA/Ag/Ca-alginate core in the course of fibre production. Consequently, a high-intensity band at 1090 cm^−1^ was observed in the FTIR spectrum of the PVA/Ag/Ca-alginate sample.

### 3.3. Swelling behaviour of PVA/Ca-alginate-based fibres

Possibilities for drying and re-swelling of PVA/Ag/Ca-alginate fibres were investigated, having in mind prospects for storage in potential applications as antimicrobial wound dressings. Rehydration of dry PVA/Ag/Ca-alginate fibres (approx. 460 µm in diameter) was investigated in physiological saline solution (0.9% w/v NaCl) at room temperature in order to imitate contact with wound exudates while Ag/Ca-alginate fibres (approx. 80 µm in diameter) served as a control.

**Figure 5.**
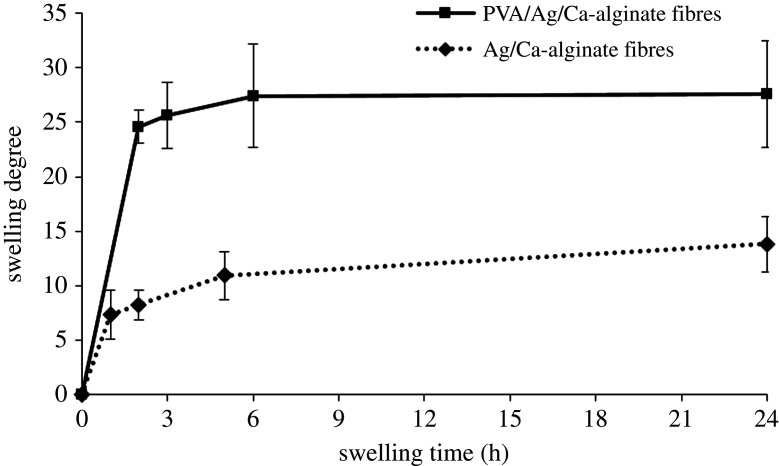
Swelling degrees of PVA/Ag/Ca-alginate and Ag/Ca-alginate fibres as functions of time in physiological saline solution (data represent average of *n* = 3).

Swelling degrees of both fibre types increased over time, reaching constant equilibrium values after approximately 24 h (figure 5). Swelling of PVA/Ag/Ca-alginate fibres was significantly higher than that of Ag/Ca-alginate fibres reaching the equilibrium degree of 28 ± 5 as compared with 14 ± 2 in the latter fibres. The increase in swelling degree in the fibres containing PVA could be explained by the fact that PVA polymer chains are more hydrophilic than alginate as reported in the literature [46,47]. Stability of the fibres was checked over 3 days having in mind the potential application as wound dressings that would be changed during that period.

### 3.4. Mechanical properties of PVA/Ca-alginate-based fibres

**Figure 6.**
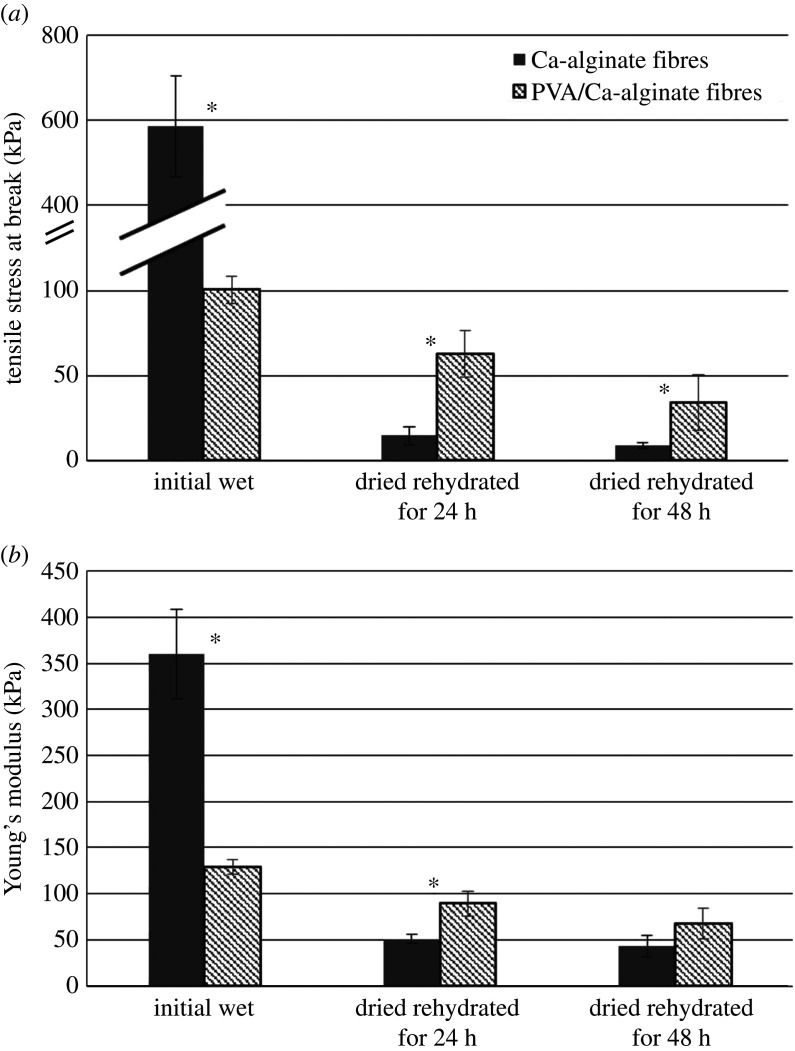
Mechanical properties of the initial wet and dried Ca-alginate and PVA/Ca-alginate fibres after rehydration for 24 and 48 h in the physiological saline solution: (*a*) tensile stress at break and (*b*) Young's modulus (asterisk designates statistically significant differences, *p* < 0.05).

Tensile stress at break (*σ*_max_) and Young's modulus (E) were determined for the produced wet Ca-alginate fibres (680 ± 60 µm) and PVA/Ca-alginate fibres (980 ± 70 µm), as well as for these fibres upon drying and rehydration for 24 and 48 h in the physiological saline solution (figure 6). After drying and rehydration for 24 h, initial Ca-alginate and PVA/Ca-alginate fibres slightly increased in size (860 ± 20 µm and 1090 ± 70 µm, respectively), while over the next 24 h of rehydration the fibre diameters stayed unchanged in both cases. Fibres with AgNPs were not assessed regarding mechanical properties as it was previously shown that incorporation of AgNPs in alginate hydrogels had negligible effects on these properties [44].

The measurements showed significantly higher values of the tensile stress at break and Young's modulus for the initial wet Ca-alginate fibres (589.8 ± 117.8 and 360.2 ± 48.3 kPa, respectively) as compared with those determined for PVA/Ca-alginate fibres (100.6 ± 40.8 and 129.5 ± 7.9 kPa, respectively). These results are in agreement with the results of higher swelling of nanocomposite PVA/Ca-alginate-based fibres implying lower interactions of PVA and alginate polymer chains than those in the Ca-alginate hydrogel. Upon drying and 24 h rehydration in physiological saline solution, mechanical properties of both fibre types decreased. However, the obtained values for the tensile stress at break and Young's modulus for PVA/Ca-alginate fibres were higher (approx. 1.8- to 4.3-fold) than the respective values for Ca-alginate fibres. The measured values further decreased after 48 h in physiological saline solution for both fibre types but still were significantly higher for PVA/Ca-alginate fibres as compared with those of Ca-alginate fibres (tensile stress at break of 34.3 ± 8.1 and 8.8 ± 1.5 kPa, respectively and Young's modulus of 89.5 ± 13.6 and 50.9 ± 4.9 kPa, respectively). Drying and rehydration of fibres is important with regard to the potential application as wound dressings, which will be distributed in dry form and slightly wetted by physiological saline before the placement on the wound. Improved mechanical properties of dried and rehydrated PVA/Ca-alginate fibres are thus relevant for the practical purposes and may be explained by interactions of PVA and alginate polymer chains during drying as it is known that hydrogel films of both polymers can be obtained by drying [48,49].

### 3.5. Antibacterial activity of nanocomposite PVA/Ag/Ca-alginate-based fibres

PVA/Ag/Ca-alginate fibres were investigated regarding antibacterial activity as an estimate of functionality in potential wound dressings. Wet and dried nanocomposite fibres, containing 5.7% w/v PVA, 1.27 ± 0.08% w/v Ca-alginate and 3.06 ± 0.08 mM AgNPs were evaluated against *S. aureus* and *E. coli* in suspensions, as common bacterial strains that cause wound infections [50]. The wet fibres used in *S. aureus* suspensions were 1150 ± 100 µm in diameter, while dried fibres were 510 ± 260 µm in diameter. The wet and dried fibres used in *E. coli* suspensions were 980 ± 90 and 390 ± 220 µm in diameter, respectively.

**Figure 7.**
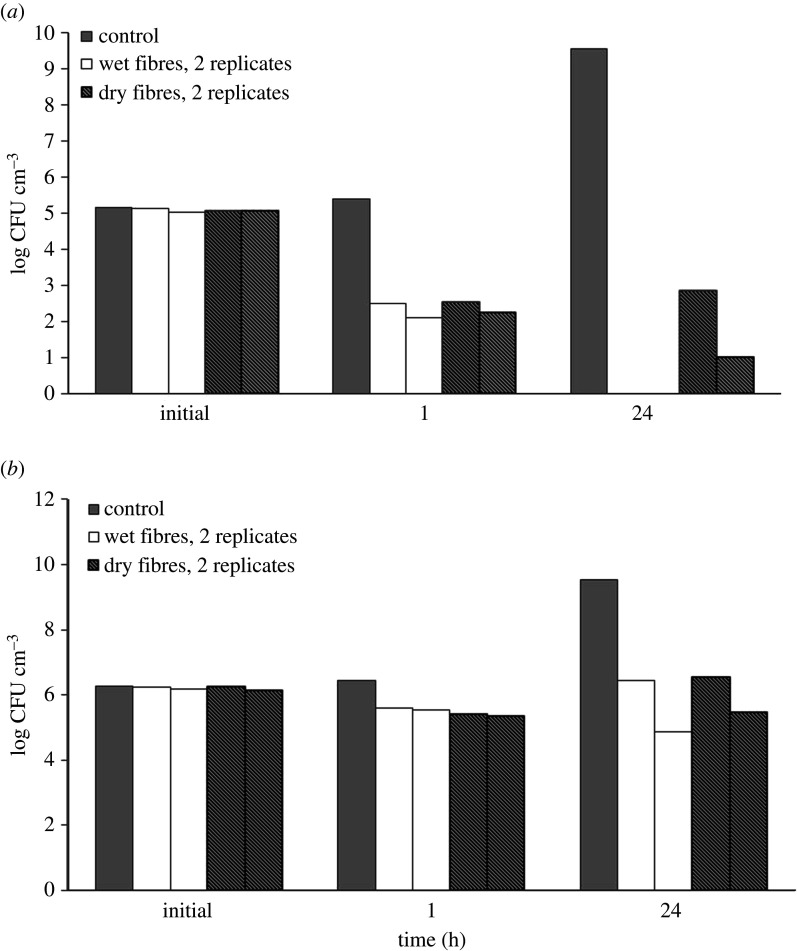
Bacterial colony number, expressed as log CFU cm^−3^, after 1 h and 24 h of incubation with wet and dry fibres in the suspension of *S. aureus* (*a*) and *E. coli* (*b*).

Both wet and dried fibres exhibited antibacterial effects against investigated bacteria due to the release of AgNPs and/or silver ions (figure 7). After 1 h of incubation, *S. aureus* concentrations in both fibre groups were lower than the initial concentration (reduction of about 3 log10-units). These values were significantly lower than those measured in the control group after 24 h of incubation (10^10^ CFU cm^−3^).

On the other hand, after 1 h of incubation, the *E. coli* concentrations in both fibre groups and the control group were the same (approx. 10^6^ CFU cm^−3^), and during the next 23 h *E. coli* concentration in both fibre groups did not significantly change, while it increased in the control group reaching the value of approximately 10^10^ CFU cm^−3^.

In order to determine the total concentration of the released silver including AgNPs, Ag^+^ and/or AgCl, which has induced the antibacterial effects, corresponding experiments were performed in physiological saline solution using wet and dried fibres. The total concentration of released silver present as AgNPs, Ag^+^ and/or AgCl in the solution after 24 h was 2.6 ± 0.9 µg cm^−3^ for both wet and dried fibres, which is approximately 2% of the initial silver content in fibres. This result is in agreement with our previous studies of silver release in physiological saline solution from dry Ag/alginate microbeads, amounting to approximately 3% of the initial microbead silver content determined after 24 h and 7 days for two alginate types [20,51]. Specifically, in our previous experimental and mathematical modelling studies of silver release kinetics from Ag/alginate microbeads in saline solution, we have shown that the release mechanism could be described by three processes: (i) diffusion of AgNPs within the hydrogel, (ii) AgNP oxidation/dissolution and reaction with chloride ions, and (iii) diffusion of formed silver-chloride species from the alginate hydrogel [51]. Furthermore, rehydration rate and swelling were shown to affect the silver release kinetics as the release from dry microbeads was lower than from wet microbeads. This finding was attributed to rapid swelling in the first case, inducing fast AgNP oxidation/dissolution, reaction with Cl^−^ and AgCl precipitation within the microbeads, which slowed diffusion from alginate matrix [51]. As PVA/Ag/Ca-alginate fibres in the present work are based on a Ca-alginate network intertwined with physically cross-linked PVA chains, the same silver release mechanism in physiological saline solution could be assumed. High swelling of this hydrogel (figure 5) probably induced rapid AgCl precipitation within the matrix and a relatively low silver release from the investigated polymer network.

The obtained results of the nanocomposite hydrogel bacteriostatic activity are in agreement with earlier studies of different systems containing AgNPs against *S. aureus* and *E. coli* [52–55]. Specifically, microbially synthesized AgNPs in *Bacillus amyloliquifaciens* at the concentration of 1.96 µg cm^−3^ exhibited bacteriostatic activity against *S. aureus*, while phytosynthesized AgNPs in a *Curcuma aromatica* tubers extract at the concentration of 7.8 µg cm^−3^ exhibited bacteriostatic activity against the same strain [53]. The minimum inhibitory concentration of AgNPs synthesized by thermal reduction using PVA as a stabilizer was 11.6 µg cm^−3^ [52], while green synthesized AgNPs in pu-erh tea leaves extract at the concentration of 7.8 µg cm^−3^ exhibited bactericidal activity against *E. coli* [55].

## 4. Conclusion

In the present study, we aimed to produce nanocomposite PVA/Ag/Ca-alginate fibres and microfibres for potential use in wound dressings. Results of our studies indicated the following conclusions.

— Microfibres and fibres with preserved AgNPs were successfully produced by extrusion with or without stretching, respectively, followed by freezing–thawing cycles.
— Detailed FTIR analyses indicate that the addition of both AgNPs and PVA polymer chains had a balanced overall contribution to the spatial arrangement of the Ca-alginate structure. Specifically, a PVA/Ag/Ca-alginate core is formed with a pendant PVA fragment at the surface.
— The presence of hydrophilic PVA polymer chains in fibres led to an increase in swelling degree and a decrease in mechanical properties as compared with Ca-alginate fibres. However, upon drying and rehydration, PVA/Ca-alginate fibres exhibited better mechanical properties as compared with those of Ca-alginate fibres, due to possible additional PVA gelation during drying.
— Nanocomposite PVA/Ag/Ca-alginate fibres, both in wet and dried forms, demonstrated antibacterial activity against *S. aureus* and *E. coli* strains at the total released silver concentration of 2.6 µg cm^−3^.

The obtained results indicate that the produced nanocomposite PVA/Ag/Ca-alginate fibres could be very attractive for wound treatments due to increased swelling and sorption capacity with better mechanical properties as compared with Ca-alginate fibres and the release of AgNPs and/or Ag^+^ inducing antibacterial activity.

## Data Availability

Data are available from the Dryad Digital Repository: https://doi.org/10.5061/dryad.79cnp5hww [56]. The data are provided in the electronic supplementary material [57].
